# Prognostic Factors in Prostate Cancer Associated with Ulcerative Colitis

**DOI:** 10.3390/jcm13051392

**Published:** 2024-02-28

**Authors:** Motoki Kaneko, Yasuhiro Kanatani, Hirohiko Sato, Masaya Sano, Erika Teramura, Jin Imai, Mia Fujisawa, Masashi Matsushima, Hidekazu Suzuki

**Affiliations:** 1 Division of Gastroenterology, Department of Internal Medicine, School of Medicine, Tokai University, 143 Shimokasuya, Isehara 259-1193, Japan; km874753@tsc.u-tokai.ac.jp (M.K.); sh580464@tsc.u-tokai.ac.jp (H.S.); m-sano@tokai.ac.jp (M.S.);; 2 Department of Clinical Pharmacology, School of Medicine, Tokai University, 143 Shimokasuya, Isehara 259-1193, Japan; 3 Department of Clinical Health Science, School of Medicine, Tokai University, 143 Shimokasuya, Isehara 259-1193, Japan

**Keywords:** ulcerative colitis, prostate cancer, older patient, biologics, pancolitis

## Abstract

Ulcerative colitis (UC) has been associated with increased prostate cancer (PCa) risk. However, the mechanisms underlying UC and increased PCa risk remain unclear, and research on this topic is scarce in Japan. We have investigated whether UC is associated with PCa risk in the Japanese population and the risk factors related to PCa among older UC patients. This retrospective single-center cohort study was conducted between January 2010 and April 2022. A total of 68 cases were analyzed, and 9 cases of PCa were observed (13.2%). PCa occurred more frequently in the adult-onset group (8/40, 20.0%) than in the older-onset group with UC (1/28; 3.57%). No significant differences were observed between immunosuppressive therapies and PCa in patients, excluding those with pancolitis-type UC. PCa occurred more frequently in the pancolitis type, and the biologics group had no PCa cases, but the difference was not statistically significant (*p* = 0.07). This study suggests that pancolitis type and UC onset in middle-aged patients may be risk factors and found that biologics potentially suppress PCa development.

## 1. Introduction

Inflammatory bowel disease (IBD) is a chronic inflammatory disease characterized by ulcerative colitis (UC) and Crohn’s disease (CD) caused by dysregulated immune responses. In recent decades, its incidence has been increasing not only in North America, Oceania, and many countries in Europe, Japan, China, India, but also in newly industrialized regions such as Southeast Asia, South America, the Middle East, and Africa [1,2,3], becoming a global disease that imposes significant social and economic burdens on healthcare systems and governments [4]. In addition to the epidemiological increase, there are also many cases of gastrointestinal malignancy [5] and extraintestinal malignancies [6,7], which could also pose a social burden while malignancy management is one the major goals of IBD treatment. Although the mechanisms of IBD and carcinogenesis are not understood, chronic inflammation contributes to intestinal malignancy in IBD and has been reported to be associated with extraintestinal malignancies [8,9].

Among UC patients, chronic inflammation can lead to the occurrence of colorectal cancer (CRC). CRC has attracted considerable attention from IBD physicians. Eaden has reported a cumulative probability of CRC in patients with UC of 2%, 8%, and 18% after 10, 20, and 30 years, respectively [10]. However, several recent population- and referral-based studies suggest that this risk may decline over time or be lower than previously accepted [11]. This may be due to the emergence of molecular targeted drugs, such as biologics and small molecular agent compounds, over the past 20 years, which have made it possible to control chronic inflammation [12].

In contrast, extraintestinal malignancies may be partially caused by chronic systemic inflammation and/or immunosuppressive therapy used to treat IBD [13]. The most consistent evidence for increased cancer risk among people with IBD exists for those treated with thiopurine medications (at risk for lymphoma [14,15,16], and non-melanoma skin cancer [17,18,19,20]), and among those treated with anti-tumor necrosis factor (anti-TNF) biological therapy (at risk for melanoma [21]). However, the risk of other extraintestinal cancers varies across studies and requires further investigation. Recently, the correlation between IBD and prostate cancer (PCa) has received widespread attention. While several of these studies have conflicting results, many have reported that, at least among patients with IBD, UC may contribute to the development of PCa [22,23,24,25,26,27,28]. However, data on the risks and mechanisms behind UC contributions to PCa development are limited. Therefore, epidemiological studies are required to determine whether the relationship between UC and the development of PCa are mediated by inflammation or immunosuppressive agents.

PCa is the most frequently diagnosed cancer among men in more than half of all countries worldwide (112 of 185 countries/territories), with an estimated 1.4 million new cases in 2020. Furthermore, PCa is the leading cause of cancer-related deaths among men in a quarter of the world’s countries (48 of 185 countries), with an estimated 375,000 deaths in 2020 [29]. PCa screening poses a dilemma in reducing the spread and mortality risk of PCa by over-diagnosing some low-risk tumors that may not have caused harm. Because treatment has potential side effects, it is critical that not all patients with PCa receive aggressive treatment [30].

The guidelines in both the United States and Europe acknowledge the benefits of identifying the risk factors for PCa to better counsel men about the use of prostate-specific antigen (PSA)-based screening [31,32]. Therefore, we examined the risk factors for PCa in patients with UC who are expected to have a high incidence of PCa in Japan. In particular, we performed a single-center cohort study targeting older patients with UC and those with older-onset UC. The number of these cases is currently on the rise [33,34,35], and a higher incidence of PCa than adult UC patients is expected.

Our hypotheses were as follows: (1) The longer the UC disease duration, the higher the incidence rate of PCa. (2) Similar to CRC occurrence, when comparing disease distribution, the incidence of PCa is high among pancolitis types of UC. (3) Since the rectum and prostate are anatomically adjacent, cases of severe inflammation in the rectum may directly cause inflammation in the prostate, increasing the incidence of PCa. (4) Systemic inflammation caused by UC may involve PCa development by causing prostatic inflammation indirectly, and the use of immunosuppressants influences the development of PCa.

## 2. Materials and Methods

### 2.1. Study Design and Subjects

A single-center cohort study was conducted on 68 male patients with UC aged ≥65 years who visited Tokai University Hospital from January 2013 to April 2022, received care from a gastroenterologist, and underwent regular follow-up observations. The inclusion criterion was a diagnosis of UC based on clinical, endoscopic, and histological criteria according to the Japanese Research Group for Intractable Inflammatory Bowel Disorders, Ministry of Health, Labor, and Welfare. The disease duration was set at ≥1 year. On average, patients underwent regular surveillance examinations with lower gastrointestinal endoscopy every 1–2 years.

**Figure 1:**
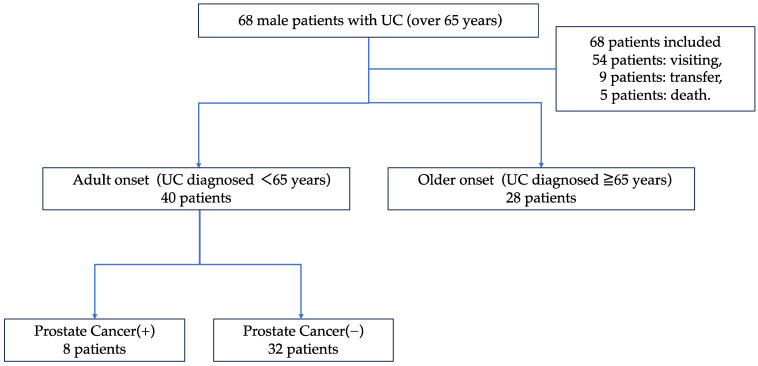
Flowchart of this study.

Figure 1 presents a flowchart of the study. Of the 68 entries, 54 patients were attending the hospital at the time of cohort enrollment, 9 patients were transferred, and 5 patients died. Data at the time of transfer were recorded for patients who were transferred to another hospital. The patients were divided into a UC adult-onset group (non-older-onset group, 40 patients were under 65 years old at the time of UC diagnosis) and a control group of 28 patients in the older-onset group, whose age at diagnosis was >65 years. We then conducted a comparative study between the two groups with PCa incidence as the primary outcome. The definition of PCa diagnosis was that a urologist suspected PCa based on a PSA test or imaging test and confirmed this by histopathological examination.

Next, we classified and compared the 40 patients in the non-older onset group into those who developed PCa and those who did not and examined risk factors related to the development of PCa.

The research protocol was approved by the Research Ethics Committee of Tokai University Hospital (No. 22R018-001H), and the research involving human subjects was conducted in accordance with the Declaration of Helsinki.

### 2.2. Information Collection

Data were extracted from electronic medical records. The following information were extracted: personal data (age; body mass index [BMI]; smoking history; and previous medical history, including cancer), UC-related data (date of diagnosis; disease duration; disease distribution; stage classification; clinical course; history of surgery for UC and hospitalization in active UC; past endoscopic severe findings; and therapies, including immunotherapy), and PCa-related data (date of diagnosis; treatment; staging; PSA level at diagnosis; and history of PSA screening tests) (Supplementary Table S1).

We classified BMI into three classes according to the World Health Organization (WHO) definitions: underweight and normal weight (BMI: <25 kg/m^2^), overweight (BMI: 25–29.9 kg/m^2^), and obese (BMI: ≥30 kg/m^2^). The disease distribution was defined according to the Montreal classification [36]: E1, ulcerative proctitis; E2, left-sided UC; E3, pancolitis. Segmental colitis (SC) was defined as a segmental distribution not belonging to the Montreal classification system. Past endoscopic findings were defined as a Mayo endoscopic subscore (MES 3) or ulcer scar findings of the rectum.

Immunotherapies were classified and collected as corticosteroids, thiopurine (azathioprine), biologics, JAK inhibitors, and calcineurin inhibitors. As for biologics, anti-TNFα monoclonal antibodies (infliximab, adalimumab, golimumab), anti-α4β7 integrin monoclonal antibody (vedolizumab), and anti-IL-12/23 p40 monoclonal antibody (ustekinumab) were used. Tofacitinib is a JAK inhibitor, and tacrolimus is a calcineurin inhibitor. The history of immunotherapy uses before and after the onset of PCa were collected and included in the analysis (mainly, in this analysis, history of use up to PCa diagnosis was included in the analysis). History of surgery for UC and hospitalization in active UC, as well as past severe endoscopic findings, were similarly collected and included in the analysis. The occurrence of factors related to the development of PCa, such as history of smoking, type 2 diabetes mellitus [T2DM], benign prostatic hyperplasia [BPH], and PSA screening tests, were also collected before the development of PCa.

### 2.3. Statistical Analysis

Qualitative variables are expressed as numbers and percentages, while quantitative variables are expressed as mean values and standard deviations. Qualitative variables were analyzed using the chi-square and Fisher’s exact tests. Quantitative variables were analyzed using *t*-tests for non-matched couples. All statistical analyses were performed using Stata/MP ver.18.0 (Stata Corporation LLC, College Station, TX, USA). Statistical significance was defined as *p* < 0.05.

## 3. Results

In all subjects, the overall incidence rate of PCa was 9/68 (13.2%); in detail, it was 8/40 (20.0%) in the adult-onset group and 1/28 (3.57%) in the older-onset group, showing a trend toward higher incidence in the adult-onset group than in the older-onset group, but not a significant difference (*p* = 0.07) (Table 1).

Age, age at UC diagnosis, and UC disease duration were significantly different; however, no significant differences were found in the prognostic factors related to PCa development between the two groups.

In this comparative analysis, all eight patients with PCa in the adult-onset group were diagnosed with PCa after being diagnosed with UC. The duration from UC to PCa diagnosis was 15.1 ± 7.0 years, and the PSA value at PCa diagnosis was 15.2 ± 9.0 ng/mL. In contrast, one patient in the older-onset group was diagnosed with PCa before being diagnosed with UC. Although there was a marginally significant difference in the incidence of PCa, no significant differences were observed in the incidence of other cancers (Table 2).

Furthermore, among the patients who developed PCa, none had concurrent CRC.

Next, we classified the adult-onset group, which had a high incidence rate of PCa compared to PCa and non-PCa groups and compared the risk factors for PCa. (Table 3) Consequently, there was a significant difference between only age. Therefore, we focused on disease distribution in UC. Regarding disease distribution, pancolitis type (E3) tended to be highly associated with PCa (6/24 cases (25%)).

**Figure 2:**
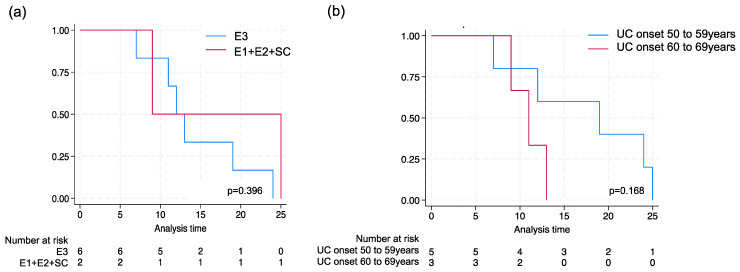
Kaplan–Meier curve for the onset of UC to PCa. (**a**) The time from onset of UC to onset of PCa was compared in two groups, E3 and E1 + E2 + SC, by inflammatory range for UC that developed PCa. (**b**) For UC with PCa, the time from onset of UC to onset of PCa was compared in the two groups of UC with onsets of UC in the 50 s and 60 s.

The Kaplan–Meier curve analysis showed that patients with pancolitis (E3) were at risk for a diagnosis of PCa at a median of 15 years (95% CI, 9–24 years) after the onset of UC (Figure 2).

Successively, we conducted a comparative study of the relationship between inflammation in UC and the development of PCa.

Because of the direct spread of inflammation from the rectum to the prostate, we compared the hospitalization history required for induction therapy for active UC and past endoscopic findings reminiscent of severe rectal inflammation, but no significant differences were found.

As the inflammation also spread indirectly from the system to the prostate, we compared the relationship between immunosuppressive therapy usage with systemic immunosuppressives, anti-inflammatory effects, and PCa development. No significant differences were observed between the immunosuppressive therapies and PCa in patients, excluding those with pancolitis type UC. PCa occurs more frequently in the pancolitis type, and the biologics group had no PCa cases, a difference that was not statistically significant difference (*p* = 0.07) (Table 4). Calcineurin inhibitors were not used before the onset of PCa in any case, and JAK inhibitors were only used in one patient in the older-onset group (who did not develop PCa). No correlation was observed with 5-ASA.

Finally, we focused on age at UC diagnosis (age at UC onset), regardless of disease distribution, and found that having a long UC disease duration was not a risk factor for PCa. When classifying the patients by age at UC onset, most patients who developed PCa (8/9 cases) experienced UC onset in their 50 s or 60 s (especially in their 50 s to early 60 s). In contrast, in the group with UC onset in their 20 s to 40 s who had a long UC disease duration, no incidence of PCa was observed. The Kaplan–Meier curve analysis showed that the median duration from UC onset to PCa diagnosis was 19 years (95% CI, 8–24 years) in the 50–59 years of UC onset and 11 years (95% CI, 9–11 years) in the 60–69 years of UC onset. If UC onset occurred in the 50 s or 60 s, there was a tendency for an older age at UC onset, and a shorter the time to PCa development.

## 4. Discussion

Although few studies have investigated the risk factors for PCa in patients with UC, this study revealed that older patients with adult-onset UC and not older-onset UC should be aware of the risk of PCa. Furthermore, although the prolonged duration of UC can be problematic, it is concerning that patients with UC onset in their 50 s to early 60 s are particularly at high risk of developing PCa. Additionally, this study suggests that the pancolitis type may be a risk factor, and immunosuppressive therapy, especially biologics, may reduce the risk of PCa.

PCa was found in 9 of 68 older patients with UC (13.2%). This was an extremely high incidence, considering that the incidence rate of PCa in the general population of Japan is 154.3 per 100,000 persons (0.15%), and the age-standardized incidence rate is 30.7 per 100,000 worldwide [37,38]. In addition, the adult-onset group, which showed a significantly longer disease duration, showed a tendency toward a higher incidence of PCa than that of the older-onset group (*p*-value = 0.07), suggesting that UC may influence the development of PCa. Each factor was compared to exclude confounding risk-related factors in PCa. Although many conflicting reports regarding acquired factors for PCa exist, smoking [39] and obesity [40] are known risk factors, and a history of T2DM [41] is a known suppressive factor; however, significant differences were not observed in these factors. Even though family history is a recognized congenital factor in PCa [42], we could not extract all the family histories in this study.

Inflammation is an acquired factor and a local cause of PCa [43]. Although no consensus has been reached, several meta-analyses have shown a significant association between prostatitis and PCa [44,45,46]. Furthermore, the anterior surface of the rectum and the peripheral zone of the prostate, a common site of PCa, are adjacent to each other [47]. We hypothesized that inflammation in the rectum due to UC may cause inflammation to spread directly to the prostate, contributing to the development of PCa. In such cases, severe rectal inflammation might be involved, with inflammation spreading beyond the Denonvillier’s fascia that separates the prostate and the front of the rectum. Therefore, we examined the hospitalization history required for induction therapy for active UC, as well as MES3 and the endoscopic findings of rectal ulcer scars, but no significant difference between the PCa and non-PCa groups was observed.

Therefore, further research must be conducted to determine the effect of inflammation of the anterior surface of the rectum on the peripheral zone of the prostate.

In contrast, systemic inflammation caused by UC is thought to indirectly cause prostate inflammation. Zhou et al. have shown that chronic intestinal inflammation has a pro-inflammatory effect on the prostate, which is associated with intraprostatic activation of pro-oncogenic signaling pathways and genomic instability [25]. In this study, we focused on the possible involvement of interleukin-6 (IL-6). IL-6 is a central regulator of the immune response in various types of inflammatory diseases similar to TNFα [48] and has also been reported to be a multifunctional cytokine regulating the growth of PCa [49].

Furthermore, IL-6 is considered a pivotal tumor promoter in colitis-associated cancer [50].

In this context, it may be necessary to pay attention to the relationship between PCa development and UC as well as the occurrence of CRC. In this study, the incidence of PCa was higher in patients with pancolitis-type UC, suggesting that widespread inflammation, including that of IL-6, may be more involved in PCa development than in other types, such as proctitis or left-sided colitis. Interestingly, none of the patients who received biologics developed PCa.

Studies on the predictors of clinical responses to biological therapy in patients with inflammatory bowel disease have focused on serum IL-6 levels. IL-6 was significantly correlated with standard inflammatory biomarkers (i.e., CRP and FC) and disease duration, which varied significantly during biological treatment. In this study, a decrease in IL-6 was observed even with biologics that do not have a direct IL-6 suppression mechanism, such as anti-TNFα (infliximab, adalimumab), ustekinumab, and vedolizumab [51].

This suggests that the long-term direct or indirect IL-6 suppressive effects of biological drugs could contribute to the suppression of PCa. Similar biologics were used in this study. In this study, PCa, dysplasia, and CRC did not overlap. This suggests that carcinogenesis of either type may not be entirely explained by IL-6; therefore, further studies are required.

Finally, the results of this study suggest that the age at the onset of UC, not prolonged UC duration, is a risk factor for PCa development. The fact that PCa occurred more frequently in patients of the adult-onset UC group than of the older-onset group suggests that long UC disease duration may be a risk factor for PCa development. However, when comparing the time of UC onset, none of the patients in the long-term disease group, with UC onset in their 20 s and 40 s, developed PCa. PCa occurrence was concentrated in patients with UC onset during their 50 s to early 60 s.

The existence of latent carcinoma (cancer that shows no clinical signs of PCa and is only confirmed at postmortem autopsy) supports this idea. In an autopsy study of young individuals, small latent cancers reportedly started occurring from the age of 30 years [52]. According to age, for Asian people in their 40 s, 50 s, 60 s, 70 s, 80 s, and 90 s, 6.3%, 17.3%, 17.7%, 25.4%, 33.2%, and 50.0% developed cancer; for Caucasian people, this was 15.0%, 26.9%, 33.3%, 35.4%, 49.0%, 91.1%; and for Black people: 24.7%, 39.6%, 56.7%, respectively, (no data are available for people over 70 years of age); this demonstrates that cancer occurrence is higher with increasing age [53].

PCa may grow slowly over several decades because the frequency of latent cancer increases with age. We assumed that being older than above middle age is a risk period for latent cancer, since the onset of UC occurs during this period, causing local or systemic inflammation in the latent cancer and contributing to the initiation or promotion of PCa development.

A limitation of this study is its small sample size. However, unlike previous studies, this study sheds light on the risk of PCa in patients with UC. It was also challenging to collect detailed family histories of older patients. However, concerning the individual cancers confirmed in patients, there were only two cases in which PCa overlapped with different cancers (PCa and brain tumor, and PCa and stomach cancer). We considered that the genetic factor characteristic of multiple primary cancers are unlikely to have influenced the epidemiological background of cancer development in this study.

## 5. Conclusions

Although the study data originated from a single institution, UC was revealed as a risk factor for PCa. Pancolitis type and age at UC onset (50 s to early 60 s) may be risk factors for PCa. In contrast, our findings suggest that the effects of prolonged anti-inflammatory biologics may reduce the risk of developing PCa. Since the study required a uniform specialist’s perspective and the follow-up of individual cases over ten years or more, limiting the study to a single institution allowed us to identify risk factors for UC-related prostate cancer. In this regard, this study is a pilot study for the next step of conducting a nationwide survey, and we plan to receive a nationwide UC database from the Ministry of Health, Labour, and Welfare to analyze further risk factors that did not statistically differ enough in this study [54].

## Figures and Tables

**Table 1:** Comparison of adult-onset and older-onset UC.

Variables	Adult Onset	Older Onset	Total	*p*-Value
Patients, *n*	40	28	68	-
Age (year, median ± SD)	73.5 ± 5.2	78.8 ± 6.6	75.6 ± 6.3	<0.05
Age at UC diagnosis (year, median ± SD)	52.3 ± 8.6	71.2 ± 4.9	60.1 ± 11.9	<0.05
UC disease duration (year, median ± SD)	21.2 ± 8.8	8.0 ± 5.0	15.7 ± 9.9	<0.05
Type E1, E2, E3	9:6:24:(1) †	5:6:17	14:12:41:(1) †	-
BMI < 25 (kg/m^2^)	33	25	58	0.51
BMI 25–29.9 (kg/m^2^)	6	3	9	0.73
BMI ≥ 30 (kg/m^2^)	1	0	1	N.S.
Smoking history, *n* (%) ‡	26 (65.0%)	20 (71.4%)	46 (67.6%)	0.61
T2DM, *n* (%)	8 (20.0%)	5 (17.9%)	13 (19.1%)	N.S.
BPH, *n* (%)	11 (27.5%)	4 (14.3%)	15 (22.1%)	0.24
History of	18 (45.0%)	9 (32.1%)	27 (39.7%)	0.32
PSA screening tests, *n* (%)				
Surgery (UC related), *n* (%)	6 (15.0%)	4 (14.3%)	10 (14.7%)	N.S.
Cancer (dysplasia/CRC-), *n* (%)	12 (30.0%)	6 (21.4%)	19 (27.9%)	0.58
Prostate cancer, *n* (%)	8 (20.0%)	1 (3.57%)	9 (13.2%)	0.07
Dysplasia/CRC, *n* (%)	3 (7.5%)	3 (10.7%)	5 (7.4%)	0.68

† segmental colitis, 1 case. ‡ 1 patient with adult-onset UC was a current smoker, and all the others were past smokers, - no dysplasia/CRC.

**Table 2:** Comparison of adult-onset and older-onset cancers.

Cancers	Adult Onset	Older Onset	Total	*p*-Value
	*n* = 40	*n* = 28	*n* = 68	
Prostate cancer	8 (20.0%)	1 (3.6%)	9 (13.2%)	0.07
Stomach cancer	2 (5.0%)	3 (10.7%)	5 (7.4%)	0.4
Tongue cancer	-	2 (7.1%)	2 (2.9%)	0.17
Thyroid cancer	1 (2.5%)	-	1 (1.5%)	N.S.
Buccal mucosa cancer	-	1 (3.6%)	1 (1.5%)	0.41
Bladder, ureteral cancer	1 (2.5%)	-	1 (1.5%)	N.S.
Blood cancer	1 (2.5%)	-	1 (1.5%)	N.S.
Brain tumor	-	1 (3.6%)	1 (1.5%)	0.41
Non-melanoma skin cancer (NMSC)	1 (2.5%)	-	1 (1.5%)	N.S.
Dysplasia/CRC	3 (7.5%)	3 (10.7%)	6 (8.8%)	0.68

Adult-onset group: Prostate and stomach cancers overlapped in one case. Blood cancer and NMSC overlapped in one case. In the older-onset group, buccal mucosa cancer, tongue cancer, and CRC overlapped in one case. Prostate cancer and brain tumor overlapped in one case.

**Table 3:** Comparison of adult-onset group with and without prostate cancer.

	Adult Onset		*p*-Value
	PCa (+)	PCa (−)	
Patients, *n*	8	32	-
Age (year, median ± SD)	78.1 ± 6.5	72.3 ± 4.1	<0.05
Age at UC diagnosis (year, median ± SD)	57.4 ± 3.4	51.0 ± 9.1	0.06
UC disease durations (year, median ± SD)	20.8 ± 6.1	21.3 ± 9.38	0.89
E1 + E2 + SC	2	14	0.33
E3	6	18	
BMI < 25 (kg/m^2^)	5	28	0.13
BMI 25–29.9 (kg/m^2^)	2	4	0.58
BMI ≥ 30 (kg/m^2^)	1	0	0.2
Smoking history, *n* (%)	4 (50.0%)	22 (68.8%)	0.42
T2DM, *n* (%)	2 (25.0%)	6 (18.8%)	0.65
BPH, *n* (%)	2 (25.0%)	9 (28.1%)	N.S.
Corticosteroid ever used, *n* (%)	2 (25.0%)	18 (56.3%)	0.24
Thiopurine ever used, *n* (%)	1 (12.5%)	11 (34.4%)	0.4
Biologics ever used, *n* (%)	0 (0.0%)	9 (28.1%)	0.16
Surgery (UC related), *n* (%)	1 (12.5%)	5 (15.6%)	N.S.
Dysplasia/CRC, *n* (%)	2 (6.25%)	2 (6.25%)	N.S.
History of hospitalization (UC related), *n* (%)	4 (50.0%)	15 (46.9%)	N.S.
Endoscopic severe findings, *n* (%)	4 (50.0%)	17 (53.1%)	N.S.

Immunosuppressive therapies were defined as prior use before PCa diagnosis.

**Table 4:** Relationship between inflammatory extent of UC and therapy selection and prostate cancer.

			Adult Onset (*n* = 40)							
			E1 + E2 + SC (*n* = 16)				E3 (*n* = 24)			
Therapy for UC			PCa (+) *n* = 2	PCa (−) *n* = 14	Ratio	*p*-Value	PCa (+) *n* = 6	PCa (−) *n* = 18	Ratio	*p*-Value
Corticosteroid	+	*n* = 20	0	6	0%	0.24	2	12	17%	0.15
	−	*n* = 20	2	8	20%		4	6	40%	
Thiopurine	+	*n* = 12	0	6	0%	0.24	1	5	17%	0.59
	−	*n* = 28	2	8	20%		5	13	28%	
Biologics	+	*n* = 9	0	2	0%	0.57	0	7	0%	0.07
	−	*n* = 31	2	12	17%		6	11	35%	

## Data Availability

Data are contained within the article and Supplementary Materials.

## Supplementary Materials

The following supporting information can be downloaded at: https://www.mdpi.com/article/10.3390/jcm13051392/s1, Table S1: Ulcerative colitis survey items.

## References

[1] Ng S.C., Shi H.Y., Hamidi N., Underwood F.E., Tang W., Benchimol E.I., Panaccione R., Ghosh S., Wu J.C.Y., Chan F.K.L. (2017). Worldwide incidence and prevalence of inflammatory bowel disease in the 21st century: A systematic review of population-based studies. Lancet.

[2] Park S.H. (2022). Update on the epidemiology of inflammatory bowel disease in Asia: Where are we now?. Intest. Res..

[3] Yamazaki M., Chung H., Xu Y., Qiu H. (2023). Trends in the prevalence and incidence of ulcerative colitis in Japan and the US. Int. J. Color. Dis..

[4] Kaplan G.G. (2015). The global burden of IBD: From 2015 to 2025. Nat. Rev. Gastroenterol. Hepatol..

[5] Faye A.S., Holmer A.K., Axelrad J.E. (2022). Cancer in Inflammatory Bowel Disease. Gastroenterol. Clin. N. Am..

[6] Yadav S., Singh S., Harmsen W.S., Edakkanambeth V.J., Tremaine W.J., Loftus E.V. (2015). Effect of Medications on Risk of Cancer in Patients With Inflammatory Bowel Diseases: A Population-Based Cohort Study from Olmsted County, Minnesota. Mayo Clin. Proc..

[7] Pedersen N., Duricova D., Elkjaer M., Gamborg M., Munkholm P., Jess T. (2010). Risk of extra-intestinal cancer in inflammatory bowel disease: Meta-analysis of population-based cohort studies. Am. J. Gastroenterol..

[8] Axelrad J.E., Lichtiger S., Yajnik V. (2016). Inflammatory bowel disease and cancer: The role of inflammation, immunosuppression, and cancer treatment. World J. Gastroenterol..

[9] Trikha M., Corringham R., Klein B., Rossi J.F. (2003). Targeted anti-interleukin-6 monoclonal antibody therapy for cancer: A review of the rationale and clinical evidence. Clin. Cancer Res..

[10] Eaden J.A., Abrams K.R., Mayberry J.F. (2001). The risk of colorectal cancer in ulcerative colitis: A meta-analysis. Gut.

[11] Zisman T.L., Rubin D.T. (2008). Colorectal cancer and dysplasia in inflammatory bowel disease. World J. Gastroenterol..

[12] Lasa J.S., Olivera P.A., Danese S., Peyrin-Biroulet L. (2022). Efficacy and safety of biologics and small molecule drugs for patients with moderate-to-severe ulcerative colitis: A systematic review and network meta-analysis. Lancet Gastroenterol. Hepatol..

[13] Mathias H., Rohatinsky N., Murthy S.K., Novak K., Kuenzig M.E., Nguyen G.C., Fowler S., Benchimol E.I., Coward S., Kaplan G.G. (2023). The 2023 Impact of Inflammatory Bowel Disease in Canada: Access to and Models of Care. J. Can. Assoc. Gastroenterol..

[14] Kotlyar D.S., Lewis J.D., Beaugerie L., Tierney A., Brensinger C.M., Gisbert J.P., Loftus E.V., Peyrin-Biroulet L., Blonski W.C., Van Domselaar M. (2015). Risk of lymphoma in patients with inflammatory bowel disease treated with azathioprine and 6-mercaptopurine: A meta-analysis. Clin. Gastroenterol. Hepatol..

[15] Beaugerie L., Brousse N., Bouvier A.M., Colombel J.F., Lémann M., Cosnes J., Hébuterne X., Cortot A., Bouhnik Y., Gendre J.P. (2009). Lymphoproliferative disorders in patients receiving thiopurines for inflammatory bowel disease: A prospective observational cohort study. Lancet.

[16] Khan N., Abbas A.M., Lichtenstein G.R., Loftus E.V., Bazzano L.A. (2013). Risk of lymphoma in patients with ulcerative colitis treated with thiopurines: A nationwide retrospective cohort study. Gastroenterology.

[17] Narous M., Nugent Z., Singh H., Bernstein C.N. (2023). Risks of Melanoma and Nonmelanoma Skin Cancers Pre- and Post-Inflammatory Bowel Disease Diagnosis. Inflamm. Bowel Dis..

[18] Abbas A.M., Almukhtar R.M., Loftus E.V., Lichtenstein G.R., Khan N. (2014). Risk of melanoma and non-melanoma skin cancer in ulcerative colitis patients treated with thiopurines: A nationwide retrospective cohort. Am. J. Gastroenterol..

[19] Kopylov U., Vutcovici M., Kezouh A., Seidman E., Bitton A., Afif W. (2015). Risk of Lymphoma, Colorectal and Skin Cancer in Patients with IBD Treated with Immunomodulators and Biologics: A Quebec Claims Database Study. Inflamm. Bowel Dis..

[20] Long M.D., Herfarth H.H., Pipkin C.A., Porter C.Q., Sandler R.S., Kappelman M.D. (2010). Increased risk for non-melanoma skin cancer in patients with inflammatory bowel disease. Clin. Gastroenterol. Hepatol..

[21] Esse S., Mason K.J., Green A.C., Warren R.B. (2020). Melanoma risk in patients treated with biologic therapy for common inflammatory diseases: A systematic review and meta-analysis. JAMA Dermatol..

[22] Meyers T.J., Weiner A.B., Graff R.E., Desai A.S., Cooley L.F., Catalona W.J., Hanauer S.B., Wu J.D., Schaeffer E.M., Abdulkadir S.A. (2020). Association between inflammatory bowel disease and prostate cancer: A large-scale, prospective, population-based study. Int. J. Cancer.

[23] Jung Y.S., Han M., Park S., Kim W.H., Cheon J.H. (2017). Cancer Risk in the Early Stages of Inflammatory Bowel Disease in Korean Patients: A Nationwide Population-based Study. J. Crohns. Colitis.

[24] So J., Tang W., Leung W.K., Li M., Lo F.H., Wong M.T.L., Sze A.S.F., Leung C.M., Tsang S.W.C., Shan E.H.S. (2017). Cancer Risk in 2621 Chinese Patients with Inflammatory Bowel Disease: A Population-based Cohort Study. Inflamm. Bowel Dis..

[25] Zhou B.G., Yu Q., Jiang X., Mei Y.Z., Ding Y.B., Wang M. (2023). Association between inflammatory bowel disease and risk of incident prostate cancer: A systematic review and meta-analysis of cohort studies. Int. J. Colorectal. Dis..

[26] Zhang C., Liu S., Peng L., Wu J., Zeng X., Lu Y., Shen H., Luo D. (2021). Does inflammatory bowel disease increase the risk of lower urinary tract tumors: A meta-analysis. Transl. Androl. Urol..

[27] Ge Y., Shi Q., Yao W., Cheng Y., Ma G. (2020). The association between inflammatory bowel disease and prostate cancer risk: A meta-analysis. Prostate Cancer Prostatic Dis..

[28] Chen M., Yuan C., Xu T. (2020). An increase in prostate cancer diagnosis during inflammatory bowel disease: A systematic review and meta-analysis. Clin. Res. Hepatol. Gastroenterol..

[29] Bergengren O., Pekala K.R., Matsoukas K., Fainberg J., Mungovan S.F., Bratt O., Bray F., Brawley O., Luckenbaugh A.N., Mucci L. (2023). 2022 Update on Prostate Cancer Epidemiology and Risk Factors-A Systematic Review. Eur. Urol..

[30] Loeb S., Bjurlin M.A., Nicholson J., Tammela T.L., Penson D.F., Carter H.B., Carroll P., Etzioni R. (2014). Overdiagnosis and overtreatment of prostate cancer. Eur. Urol..

[31] Grossman D.C., Curry S.J., Owens D.K., Bibbins-Domingo K., Caughey A.B., Davidson K.W., Doubeni C.A., Ebell M., Epling J.W., US Preventive Services Task Force (2018). Screening for Prostate Cancer: US Preventive Services Task Force Recommendation Statement. JAMA.

[32] Gandaglia G., Albers P., Abrahamsson P.A., Briganti A., Catto J.W.F., Chapple C.R., Montorsi F., Mottet N., Roobol M.J., Sønksen J. (2019). Structured Population-based Prostate-specific Antigen Screening for Prostate Cancer: The European Association of Urology Position in 2019. Eur. Urol..

[33] Takahashi H., Matsui T., Hisabe T., Hirai F., Takatsu N., Tsurumi K., Kanemitsu T., Sato Y., Kinjyo K., Yano Y. (2014). Second peak in the distribution of age at onset of ulcerative colitis in relation to smoking cessation. J. Gastroenterol. Hepatol..

[34] Shi H.Y., Chan F.K., Leung W.K., Li M.K., Leung C.M., Sze S.F., Ching J.Y., Lo F.H., Tsang S.W., Shan E.H. (2016). Natural History of Elderly-onset Ulcerative Colitis: Results from a Territory-wide Inflammatory Bowel Disease Registry. J. Crohns. Colitis.

[35] Song E.M., Lee H.S., Park S.H., Kim G.U., Seo M., Hwang S.W., Yang D.H., Kim K.J., Byeon J.S., Myung S.J. (2018). Clinical characteristics and long-term prognosis of elderly onset ulcerative colitis. J. Gastroenterol. Hepatol..

[36] Satsangi J., Silverberg M.S., Vermeire S., Colombel J.F. (2006). The Montreal classification of inflammatory bowel disease: Controversies, consensus, and implications. Gut.

[37] Nationwide Cancer Incidence Data (2016~2019)/National Cancer Registry/Cancer Statistics Cancer Information Service, National Cancer Center, Japan (National Cancer Registry, Ministry of Health, Labour and Welfare). https://ganjoho.jp/reg_stat/statistics/data/dl/index.html#a14.

[38] World Health Organization (2020). Globocan. http://globocan.iarc.fr/Default.aspx.

[39] Al-Fayez S., El-Metwally A. (2023). Cigarette smoking and prostate cancer: A systematic review and meta-analysis of prospective cohort studies. Tob. Induc. Dis..

[40] Tzenios N., Tazanios M.E., Chahine M. (2022). The impact of body mass index on prostate cancer: An updated systematic review and meta-analysis. Medicine.

[41] Amadou A., Freisling H., Jenab M., Tsilidis K.K., Trichopoulou A., Boffetta P., Van Guelpen B., Mokoroa O., Wilsgaard T., Kee F. (2021). Prevalent diabetes and risk of total, colorectal, prostate and breast cancers in an ageing population: Meta-analysis of individual participant data from cohorts of the CHANCES consortium. Br. J. Cancer.

[42] Albright F., Stephenson R.A., Agarwal N., Teerlink C.C., Lowrance W.T., Farnham J.M., Albright L.A. (2015). Prostate cancer risk prediction based on complete prostate cancer family history. Prostate.

[43] Sfanos K.S., De Marzo A.M. (2012). Prostate cancer and inflammation: The evidence. Histopathology.

[44] Langston M.E., Horn M., Khan S., Pakpahan R., Doering M., Dennis L.K., Sutcliffe S. (2019). A Systematic Review and Meta-analysis of Associations between Clinical Prostatitis and Prostate Cancer: New Estimates Accounting for Detection Bias. Cancer Epidemiol. Biomark. Prev..

[45] Zhang L., Wang Y., Qin Z., Gao X., Xing Q., Li R., Wang W., Song N., Zhang W. (2020). Correlation between Prostatitis, Benign Prostatic Hyperplasia and Prostate Cancer: A systematic review and Meta-analysis. J. Cancer.

[46] Jung G., Kim J.K., Kim H., Lee J., Hong S.K. (2022). The association between prostatitis and risk of prostate cancer: A National Health Insurance Database study. World J. Urol..

[47] Lindsey I., Guy R.J., Warren B.F., Mortensen N.J. (2000). Anatomy of Denonvilliers’ fascia and pelvic nerves, impotence, and implications for the colorectal surgeon. Br. J. Surg..

[48] Aliyu M., Zohora F.T., Anka A.U., Ali K., Maleknia S., Saffarioun M., Azizi G. (2022). Interleukin-6 cytokine: An overview of the immune regulation, immune dysregulation, and therapeutic approach. Int. Immunopharmacol..

[49] Culig Z., Puhr M. (2012). Interleukin-6: A multifunctional targetable cytokine in human prostate cancer. Mol. Cell Endocrinol..

[50] Li W., Zhao T., Wu D., Li J., Wang M., Sun Y., Hou S. (2022). Colorectal Cancer in Ulcerative Colitis: Mechanisms, Surveillance and Chemoprevention. Curr. Oncol..

[51] Waldner M.J., Neurath M.F. (2014). Master regulator of intestinal disease: IL-6 in chronic inflammation and cancer development. Semin. Immunol..

[52] Sakr W.A., Haas G.P., Cassin B.F., Pontes J.E., Crissman J.D. (1993). The frequency of carcinoma and intraepithelial neoplasia of the prostate in young male patients. J. Urol..

[53] Rebbeck T.R., Haas G.P. (2014). Temporal trends and racial disparities in global prostate cancer prevalence. Can. J. Urol..

[54] Kanatani Y., Tomita N., Sato Y., Eto A., Omoe H., Mizushima H. (2017). National Registry of Designated Intractable Diseases in Japan: Present Status and Future Prospects. Neurol. Med. Chir..

