# Supplementary material for: Prognostic Factors in Prostate Cancer Associated with Ulcerative Colitis
Source: J Clin Med. 2024 Feb 28;13(5):1392. doi: 10.3390/jcm13051392 (PMC10932459; doi:10.3390/jcm13051392)
Supplement: Supplementary file 1 [file jcm-13-01392-s001.zip › jcm-2853548-supplementary.pdf]

Supplemental material.

Table 1. Ulcerative colitis survey items

Item	Option
Date of birth	YYYY(AD)
Age at cohort entry	
Age at UC diagnosis	
Year at UC diagnosis	YYYY(AD)
Duration of UC	
Disease distribution	1:proctitis 2:left sided 3:pancolitis 4:segmental colitis
Stage classification※	1:remission stage 2:active stage 3:post-total colectomy
Partial Mayo Score	
Clinical course	1:first attack type 2:relapse-remitting type 3:chronic continuous type
Extraintestinal complications	1:Yes 2:No
Details of extraintestinal complications	Describe complications
BMI	1: <25 2: 25-29.9 3: ≥ 30
Smoking	1:Yes 2:past smoking 3:No
Past history: Type 2 diabetes mellitus (T2DM)	1:Yes 2:No
Past history: Benign prostatic hyperplasia (BPH)	1:Yes 2:No

Past history: PSA screening (prior to PCa occurrence)	1:Yes 2:No
PSA level at PCa diagnosis	
Age at PCa diagnosis	
Year at PCa diagnosis	YYYY(AD)
5-aminosalicylic acids(5-ASA) use	1:Yes 2:No
5-ASA Medication Details	1: Salazosulfapyridine<br>2: Mesalazine<br>3: Salazosulfapyridine+Mesalazine
Suppository/enema administration	1:Yes 2:No
Medications used in suppository/enema administration	1: Mesalazine 2: Budesonide<br>3: Mesalazine+Budesonide
Corticosteroid use with remission induction for UC	1:Yes 2:No
Corticosteroid use	1:Yes 2:No
Corticosteroid use up to PCa diagnosis	1:Yes 2:No
Corticosteroid usage (max/day)	
Corticosteroid usage: year at initiation use	YYYY(AD)
Thiopurine use with maintenance therapy for UC	1:Yes 2:No
Thiopurine use	1:Yes 2:No
Thiopurine use up PCa diagnosis	1:Yes 2:No
Thiopurine: year at initiation use	YYYY(AD)
Calcineurin inhibitor use with remission induction for UC	1:Yes 2:No
Calcineurin inhibitor use	1:Yes 2:No

Calcineurin inhibitor use up to PCa diagnosis	1:Yes 2:No
Calcineurin inhibitor: year at initiation use	YYYY(AD)
Molecular targeted drug use	1:Yes 2:No
Molecular targeted drug use up to PCa diagnosis	1:Yes 2:No
Molecular targeted drug: year at initiation use	YYYY(AD)
Molecularly targeted drug<br>(biologics, small molecule compounds; JAK inhibitor)	1: anti-TNFα ab<br>2: anti-TNFα ab +anti-α4β7 ab<br>3: anti-TNFα ab +anti-IL-12/23 ab<br>4: anti-TNFα ab +anti-α4β7 ab +anti-IL12/23 ab<br>5: anti-α4β7 ab<br>6: JAK inhibitor
Surgery for UC	1:Yes 2:No
Surgery for UC detail	Describe detail of surgery
Surgery for UC up to PCa diagnosis	1:Yes 2:No
Hospitalization in active UC	1:Yes 2:No
Hospitalization in active UC up to PCa diagnosis	1:Yes 2:No
MES3 (endoscopically severe)	1:Yes 2:No
MES3 (endoscopically severe) up to PCa diagnosis	1:Yes 2:No
Ulcer scar (Rectum)	1:Yes 2:No
Ulcer scar (Rectum) up to PCa diagnosis	1:Yes 2:No
ACCI (CCI by age-adjusted)	
UCCI (Updated CCI)	

Cancer

Prostate cancer (PCa)	1:Yes 2:No
Stomach cancer	1:Yes 2:No
Tongue cancer	1:Yes 2:No
Thyroid cancer	1:Yes 2:No
Buccal mucosa cancer	1:Yes 2:No
Bladder, Ureteral cancer	1:Yes 2:No
Blood cancer	1:Yes 2:No
Brain tumor	1:Yes 2:No
Non-melanoma skin cancer (NMSC)	1:Yes 2:No
Dysplasia/Colorectal cancer (CRC)	1:Yes 2:No

Previous medical history

Hypertension	1:Yes 2:No
Dyslipidemia	1:Yes 2:No
Type 2 diabetes mellitus (T2DM)	1:Yes 2:No
Bronchial asthma	1:Yes 2:No
Primary biliary cirrhosis	1:Yes 2:No
Primary sclerosing cholangitis	1:Yes 2:No
Interstitial pneumonia	1:Yes 2:No
Chronic kidney disease	1:Yes 2:No

Anal abscess	1:Yes 2:No
Nephrolithiasis	1:Yes 2:No

※ Remission stage is defined as ≦1 using “Partial Mayo Score (eliminated endoscopy)”.

MES: Mayo endoscopic sub-score

CCI: Charlson Comorbidity index
